# Near Isometric Biomass Partitioning in Forest Ecosystems of China

**DOI:** 10.1371/journal.pone.0086550

**Published:** 2014-01-22

**Authors:** Dafeng Hui, Jun Wang, Weijun Shen, Xuan Le, Philip Ganter, Hai Ren

**Affiliations:** 1 Department of Biological Sciences, Tennessee State University, Nashville, Tennessee, United States of America; 2 Key Laboratory of Vegetation Restoration and Management of Degraded Ecosystems, South China Botanical Garden, Chinese Academy of Sciences, Guangzhou, China; Beijing Forestry University, China

## Abstract

Based on the isometric hypothesis, belowground plant biomass (M_B_) should scale isometrically with aboveground biomass (M_A_) and the scaling exponent should not vary with environmental factors. We tested this hypothesis using a large forest biomass database collected in China. Allometric scaling functions relating M_B_ and M_A_ were developed for the entire database and for different groups based on tree age, diameter at breast height, height, latitude, longitude or elevation. To investigate whether the scaling exponent is independent of these biotic and abiotic factors, we analyzed the relationship between the scaling exponent and these factors. Overall M_B_ was significantly related to M_A_ with a scaling exponent of 0.964. The scaling exponent of the allometric function did not vary with tree age, density, latitude, or longitude, but varied with diameter at breast height, height, and elevation. The mean of the scaling exponent over all groups was 0.986. Among 57 scaling relationships developed, 26 of the scaling exponents were not significantly different from 1. Our results generally support the isometric hypothesis. M_B_ scaled near isometrically with M_A_ and the scaling exponent did not vary with tree age, density, latitude, or longitude, but increased with tree size and elevation. While fitting a single allometric scaling relationship may be adequate, the estimation of M_B_ from M_A_ could be improved with size-specific scaling relationships.

## Introduction

Forest biomass plays an important role in estimating forest productivity, carbon sequestration, and in sustainable forest management [1]–[8]. The partitioning of belowground biomass (M_B_) with respect to aboveground biomass (M_A_) influences both the structure and function of individual plants and of vegetation ecosystems [9]–[14]. As an important variable, biomass partitioning has been incorporated into terrestrial ecosystem models [15]–[18]. However, the influences of environmental factors and biotic factors on biomass partitioning have not been well investigated [11], [14].

Biomass partitioning can be described as a ratio of M_B_ to M_A_ (mass below ground to mass above ground, i.e. root:shoot ratio) or by using the allometric scaling function relating M_B_ and M_A_: M_B_ = a M_A_^b^, where a is a normalizing scaling constant and b represents an allometric scaling exponent [12], [14], [19], [20]. Previous studies have demonstrated that M_A_ scales nearly isometrically with respect to M_B_ (i.e. the scaling exponent b is 1, known as the isometric hypothesis) [12], [21], [22]. This isometry is derived from an analytical approach and related to how plants partition their annual total body mass into different components [12], [21], [23]. Basically, the isometric model states that leaf biomass (M_L_) scales as the ¾ power of stem biomass (M_S_), and M_S_ scales as the ¾ power of root biomass (M_R_) [12], [24]. Thus, M_A_ = M_L_+M_S_ = a_1_M_S_^3/4^+ a_2_M_R_^3/4^ = (a_2_/a_1_)^3/4^M_R_+a_2_M_R_^3/4^. If (a_2_/a_1_)^3/4^>>a_2_, M_A_ scales isometrically with respect to M_B_ (M_B_ = M_R_). The isometric hypothesis has been tested empirically and validated at individual and forest community levels [9], [12], [14], [25], [26]. For example, Niklas [10] reported that M_A_ scales as the 1.06 (95% CI 1.05∼1.08) power of M_B_ across 257 plant species. Similarly, Yang & Luo [14] found that the scaling exponent did not differ from 1 using biomass data measured during stand development in 112 forest stands.

The isometric hypothesis has also been disputed in several studies. For example, Li et al. [27] found that scaling exponents varied for various scaling relationships among the 17 major forest types in China. As a result, they claim that there is no universal scaling relationship. The influence of environmental factors on the relationship was not investigated in this study. Other studies found that different regression methods may also contribute to differences in scaling exponent estimation [11], [27], [28]. The concept of a universal scaling exponent may have been misinterpreted, as scaling exponents vary due to changes in allometric constants related to ontogeny and to differences among species or ecological settings [12], [21], [24]. In addition, many local scale studies found that biomass partitioning varies with stand age, height, soil moisture and fertility, and climate factors [29]–[32]. For example, relative allocation to root production is expected to be greater in arid than mesic environments. However, the influence of abiotic factors associated with latitude and longitude and biotic factors such as plant age and height on the relationship between M_A_ and M_B_ at large spatial scales has not been systematically investigated.

In this study, we analyzed a large forest biomass database to develop large-scale biomass partitioning patterns and identify influencing factors. The database includes branch, stem, leaf and root biomass, and other derivative information such as altitude, longitude, and elevation, covering a wide range of forests in China. Understanding the pattern and variation of biomass partitioning among age groups, tree sizes, locations, and taxonomic groups could have important implication on terrestrial ecosystem modeling. So far, most ecosystem models only consider biomass partitioning as a constant and the effects of these influencing factors on biomass partitioning have not been incorporated into terrestrial ecosystem models [16], [18], [33].

The overall aim of this study was to test whether the root:shoot ratio and scaling exponent of the allometric function vary with tree age, diameter at breast height, height, density, latitude, longitude, elevation and family. We hypothesized that: 1) As tree age and size increase, the root:shoot ratio and scaling exponent should decrease, as more biomass accumulates aboveground than belowground as trees age. Thus, ontogeny and biotic factors (i.e. tree age and size) may influence the allometric scaling relationship; 2) Conditions are drier and colder in western than eastern China, therefore the root:shoot ratio and coefficient of the scaling relationship between root and shoot biomass will decrease from west to east. Since latitude, longitude and elevation may influence precipitation and temperature, we expect that biomass partitioning will vary with these factors. To test these hypotheses, we specifically tended to (1) estimate the mean and variation of the root:shoot ratio in forest ecosystems in China; (2) develop an overall relationship between M_B_ and M_A_ using a power function; (3) investigate whether the relationship varies with biotic factors (i.e. tree age, size, and density) and abiotic factors (i.e. latitude, longitude and elevation); 4) evaluate the influences of regression methods on the scaling exponent and constant estimation; and 5) explore whether phylogeny affects root:shoot ratio and scaling relationship.

## Materials and Methods

### Ethics Statement

This study was based on a forest biomass database that includes data collected from journal publications and reports. Thus, no specific permissions were required for this study. The field studies did not involve endangered or protected species. Data will be made available upon request.

### Forest Biomass Database

**Figure 1:**
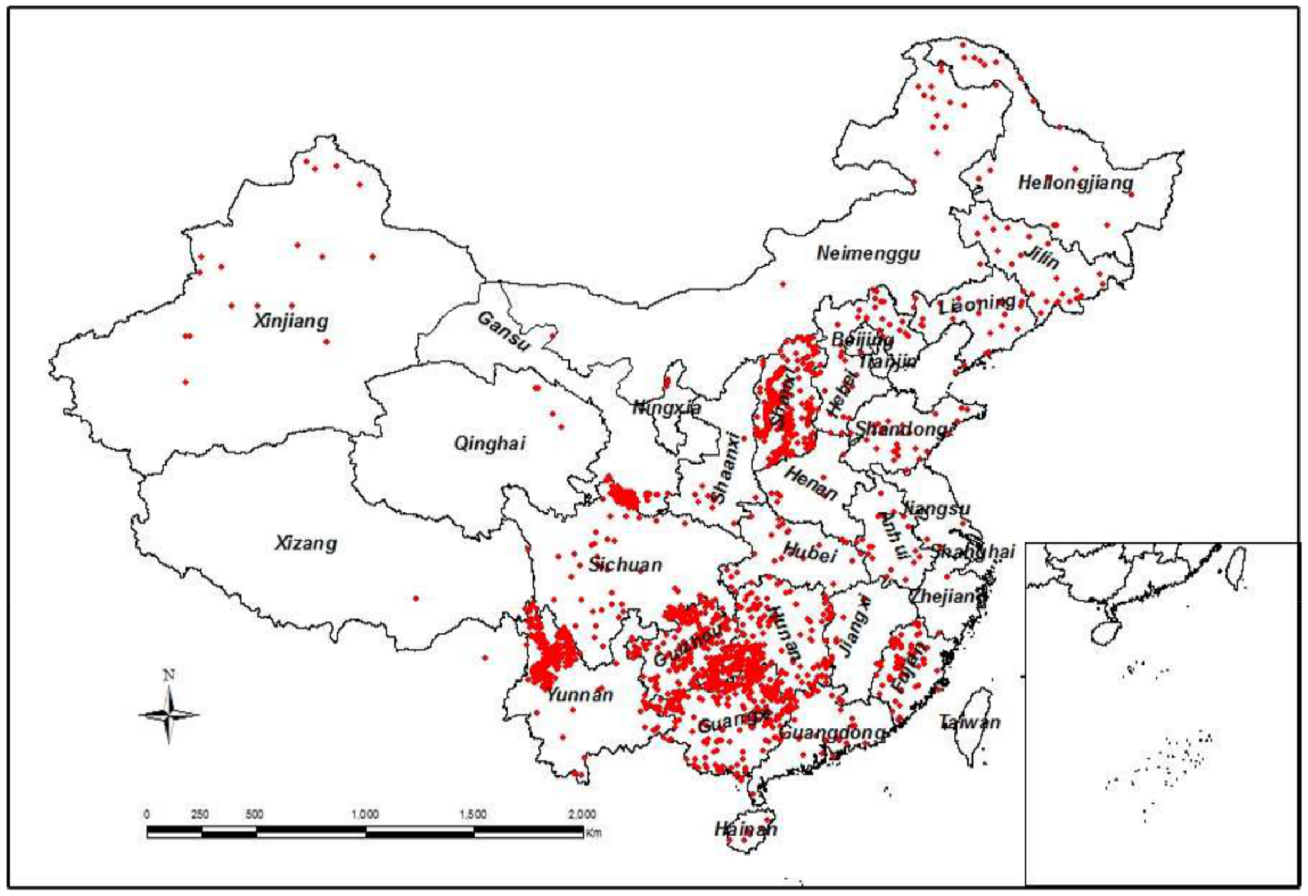
Map of sampling sites of data used in this study.

The database and the management system were constructed by Dr. Hai Ren and his colleagues at the South China Botanical Garden, Chinese Academy of Sciences, Guangzhou, China. Data were collected from journal publications and the China Forestry Inventory Reports up to 2007 (Fig. 1). In brief, this database includes 6,153 records of 550 species belonging to 75 families. The 15 families with more than 30 records each are listed in Table 1. Taxodiaceae and Pinaceae are the two most abundant families. There are 4824 evergreen trees and 1303 deciduous trees, 3276 gymnosperms and 2780 angiosperms, 4370 natural trees and 1698 planted trees. Each record in the database includes the site name, source of reference, latitude, longitude, elevation, forest origin (natural or planted), dominant species, family, leaf form (evergreen or deciduous), phylogeny type (gymnosperm or angiosperms; dicot or monocot if angiosperm), tree age, height, DBH, density of trees, leaf, stem, branch, root biomass and productivity. M_A_ was calculated as total biomass of leaf, stem and branch biomass. M_B_ was root biomass. This large database provides a unique opportunity to evaluate the relationship of productivity and biomass in Chinese forests [8] and biomass partitioning between belowground and aboveground biomass.

**Table 1:** Biomass (t ha^−1^), root:shoot ratio, and allometric scaling relationship by families, phylogeny groups and forest types.

Functional Group	n	Biomass and ratio			Allometric scaling model		
		Below- ground	Above- ground	R:S Ratio	a	b	r^2^
All data	6153	18.47	77.12	0.20	0.273	0.964	0.928
Families							
Taxodiaceae	1562	23.80	100.97	0.19	0.219	1.017	0.938
Pinaceae	1517	14.96	68.88	0.18	0.195	1.018	0.782
Fabaceae	755	21.19	84.11	0.21	0.272	0.978	0.946
Betulaceae	345	10.51	34.70	0.24	0.348	0.968	0.971
Salicaceae	349	20.07	73.05	0.22	0.241	1.015	0.804
Cupressaceae	131	10.09	53.05	0.16	0.159	1.033	0.949
Lauracea	78	10.73	53.28	0.20	0.303	0.905	0.969
Aceraceae	77	4.21	24.15	0.24	0.341	0.849*	0.957
Ulmaceae	55	8.75	29.07	0.29	0.450	0.912	0.948
Myrtaceae	51	16.92	72.31	0.20	0.237	1.003	0.941
Rosaceae	46	10.30	36.14	0.25	0.330	0.966	0.961
Theaceae	45	14.77	77.26	0.19	0.263	0.945	0.983
Tiliaceae	35	8.37	38.19	0.22	0.344	0.895	0.979
Hamamelidaceae	35	7.09	22.42	0.26	0.401	0.902	0.938
Fabacea	33	22.72	99.42	0.22	0.385	0.889	0.963
Leaf forms							
Evergreen forest	4767	19.23	81.66	0.19	0.231	0.997	0.925
Deciduous forests	1276	15.48	59.18	0.24	0.365	0.924*	0.938
Phylogenetic groups							
Gymnosperms	3235	19.52	84.96	0.19	0.191	1.038*	0.889
Angiosperms and gymnosperms mixed	95	22.21	97.99	0.21	0.518	0.808	0.888
Angiosperms	2738	17.12	67.14	0.22	0.314	0.946	0.948
Dicots	2798	17.357	68.52	0.22	0.316	0.944	0.948
Monocots	41	17.238	55.76	0.25	0.334	0.990	0.937
Forest origins							
Nature	4370	18.93	76.06	0.21	0.286	0.962	0.941
Planted	1698	17.33	79.86	0.18	0.191	1.029	0.818

n is sample size;

* indicates significantly different from 1.

Biomass measurements were conducted mostly using the “standard tree” method [8], [34], [35]. As described in Hui *et al.* [8], first, quadrats were set for each site before sampling [8]. For plantations, more than eight 10 m×10 m plots were established. For natural forests, at least twenty 10 m×10 m plots were established. Height and DBH of each individual tree and the total number of individuals in each plot were recorded. Second, five to seven standard trees for each species within a plot were selected for cutting and weighing of their component parts (stems, branches and leaves). The tree selection was based on the height and DBH measured above. Three mean size individuals, one or two smaller trees, and one or two bigger trees were selected. The stems, branches and leaves of the selected trees were weighed respectively. The coarse roots of the tree were dug, washed, separated into different root sizes based on root diameter, and weighed for fresh biomass. A sample taken from each root size was dried and weighed, and used for fresh to dry weight conversion. Total coarse biomass was estimated by adding weights of all root sizes. The fine roots were sampled using the soil block sampling method. Around each standard tree, two circles were drawn: one at a distance of that tree’s canopy size and another at half of the canopy distance. Four soil cores (3–5 cm in diameter) were taken at four directions on each circle around the stump (for a total of eight cores per tree) at different depths based on tree species, but mostly to 50 cm or deeper. Fine roots in the soil cores were washed, dried and weighed to estimate the total fine roots. Some of the stems, branches, and leaves of standard trees were taken back to the laboratory, dried, and used for calculation of the relationship between dry and fresh weights. Total biomass per plot was then computed using total tree numbers. The biomass was either measured directly by harvesting standing vegetation or estimated using the regression techniques considering DBH and/or height [8], [10], [36]–[39]. The estimation of biomass at site level from some sites might not influence the overall relationship we developed at the regional level.

### Statistical Analysis

An allometric scaling model (i.e. power function: M_B_ = aM_A_^b^) was applied to develop the relationship between M_B_ and M_A_. Several methods have been used for model parameter estimation and the pros and cons of the methods have been discussed [10], [28], [40], [41]. Reduced major axis (RMA) analysis, which estimates model parameters using a Model Type II linear regression analysis based on log-log transformed data, is the most common method applied in the literature [10], [21], [40], [42], [43]. To facilitate the comparison of model parameters with other studies, we estimated the scaling exponents and constants using RMA regression. After a log transformation, the power function was linearized and the scaling exponent b was the slope in the log-log linear regression model. We also evaluated the influence of regression methods including major axis regression (MAR), linear regression on log-log transformed data (LSR), and nonlinear regression (NLR) on scaling exponent and constant estimation [40].

**Figure 2:**
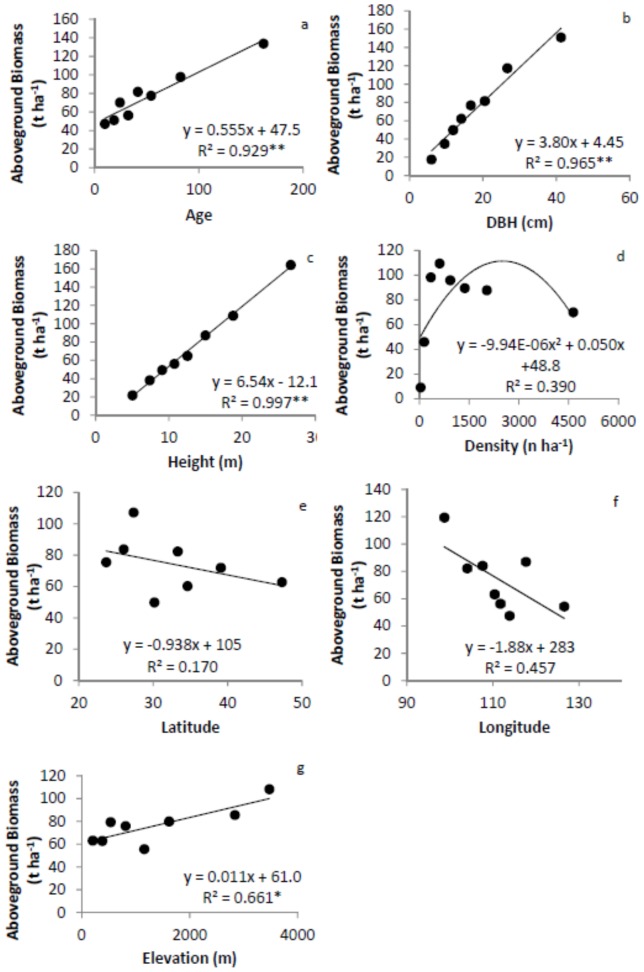
Relationships between aboveground biomass and tree age (a), diameter at breast height (DBH, b), height (c), density (d), latitude (e), longitude (f), and elevation (g). The model with the best fit among the linear, quadratic and power function models is presented. * significant at α = 0.05 level, ** significant at α = 0.01 level. Error bars are too small to be shown.

**Figure 3:**
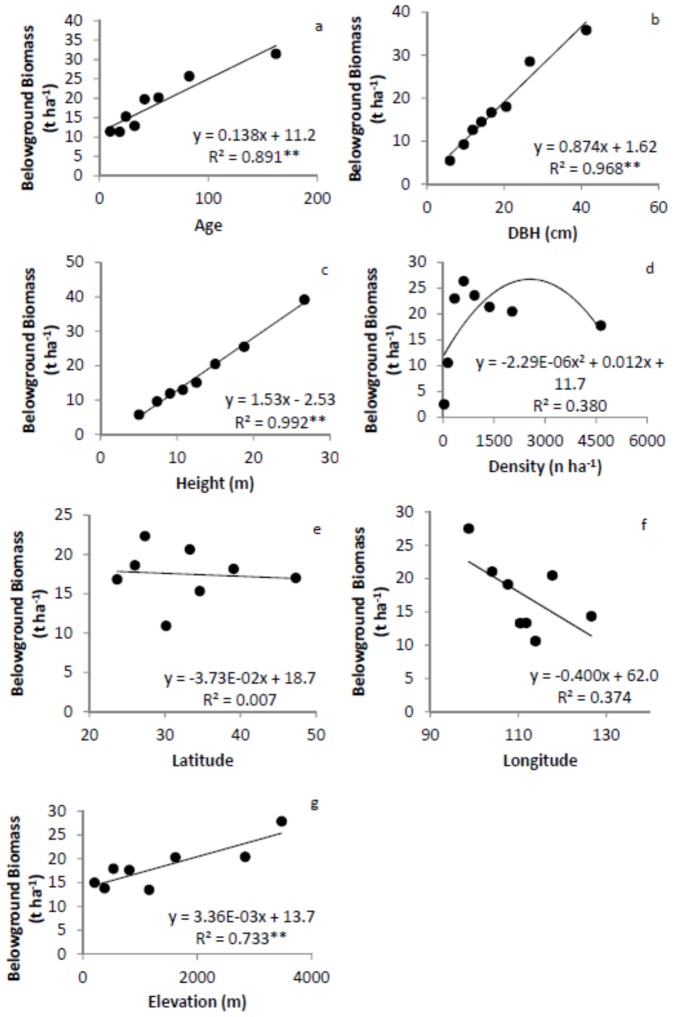
Relationships between belowground biomass and tree age (a), diameter at breast height (DBH, b), height (c), density (d), latitude (e), longitude (f), and elevation (g). The model with the best fit among the linear, quadratic and power function models is presented. ** significant at α = 0.01 level. Error bars are too small to be shown.

**Figure 4:**
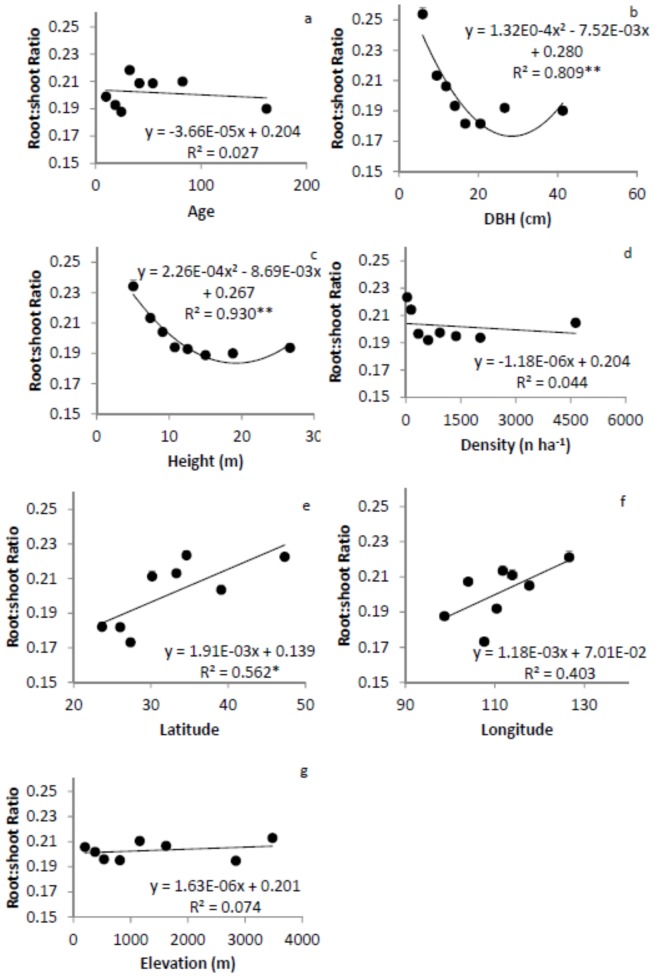
Relationships between ratio of belowground biomass (root) to aboveground (shoot) biomass and tree age (a), diameter at breast height (DBH, b), height (c), density (d), latitude (e), longitude (f), and elevation (g). The model with the best fit among linear, quadratic and power function models is presented. * significant at α = 0.05 level, ** significant at α = 0.01 level. Error bars are standard errors of the ratios.

To investigate the influence of tree age, size and geographical location on the relationship between M_B_ and M_A_, we split the entire database into 8 groups for each factor, each with a similar sample size, following Hui *et al.* [8]. We first determined the 12.5, 25, 37.5, 50, 62.5, 75, and 87.5 percentiles for each factor and grouped observations into 8 groups. For example, the corresponding ages for the percentiles above were 15, 20, 28, 35, 47, 60, and 110 years, respectively. Group 1 included 804 trees with tree age younger than or equal to 15 yr. Group 2 included 756 trees with ages older than 15 yr, but younger than or equal to 20 yr. Similarly, group 8 included 730 trees with age older than 110 yr. The same sub-sampling procedure was applied to DBH, tree height, tree density and location (i.e. latitude, longitude, and elevation). We calculated mean values for M_B_, M_A_ and root-shoot ratio, and the corresponding group variables (age, size, or location) for each group [8]. The relationships between M_A_, M_B_, and root-shoot ratio with group variables were developed for each subdivision of the data (Figs. 2, 3 and 4).

The relationship between M_B_ and M_A_ for the entire database and for each age, size, or geographical location group was developed using the allometric scaling model as described above. We further constructed the relationship of the scaling exponent and allometric constant with mean age, size, density, latitude, longitude, and elevation by fitting the best of a linear, quadratic or power function equation based on the coefficient of determination [8]. In addition, we conducted phylogenic analyses on the allometric scaling relationships across families, phylogeny, leaf forms, and forest origins. All data analyses were performed using SAS (Version 9.1, SAS Inc., Cary, NC) [44].

## Results

### Influences of Biotic and Abiotic Factors on M_A_, M_B_, and Root:Shoot Ratio

M_A_ linearly increased with tree age, DBH and height, but showed no significant relationship with density (Fig. 2). M_A_ tended to decline with increasing latitude and longitude, due to decreases in temperature and precipitation, but increased significantly with elevation. M_B_ showed very similar patterns to M_A_ (Fig. 3).

The overall root:shoot ratio was 0.202±0.00087 and ranged from 0.188 to 0.218 in age, 0.182 to 0.254 in DBH, 0.189 to 0.234 in height, 0.192 to 0.223 in density, 0.173 to 0.224 in latitude, 0.173 to 0.221 in longitude, and 0.195 to 0.213 in elevation group. There were no significant relationships between root:shoot ratio and tree age or density. The ratio decreased with DBH and height, reached low values and increased again, following quadratic equations (Fig. 4). Root:shoot ratio increased with latitude, but not with longitude or elevation.

### Influences of Biotic and Abiotic Factors on the Scaling Exponent

**Figure 5:**
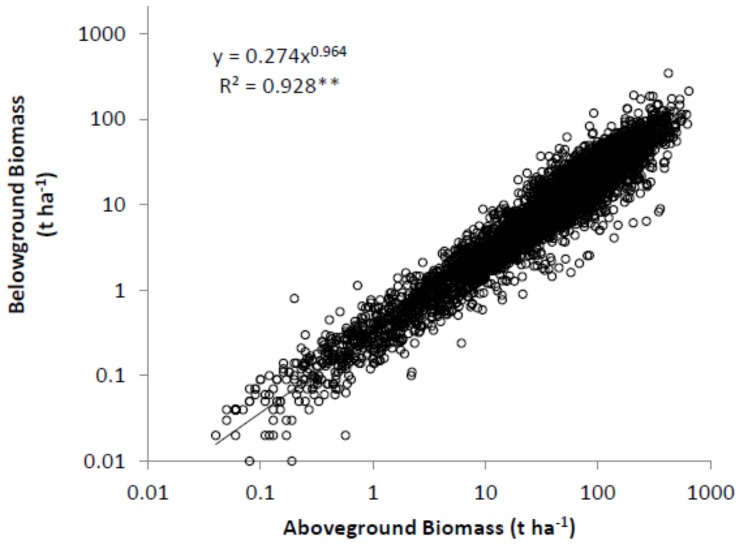
Relationship between belowground biomass and aboveground biomass of forests in China. The model is estimated using reduced major axis regression method (n = 6153). ** significant at α = 0.01 level.

**Figure 6:**
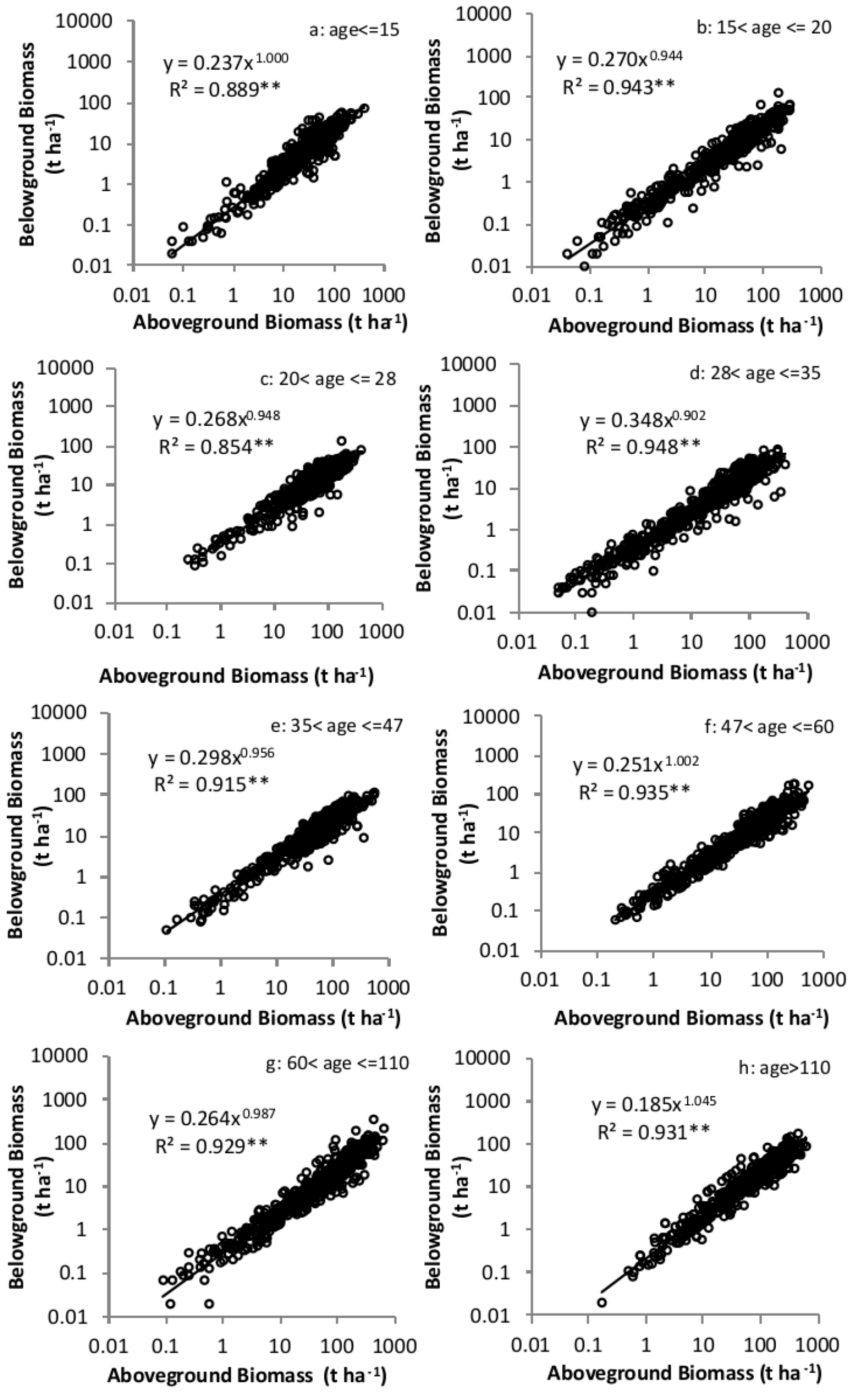
Relationships between belowground biomass and aboveground biomass for different age groups. a: age< = 15; b: 15<age< = 20; c: 20<age< = 28; d: 28<age< = 35; e: 35<age < = 47; f: 47<age < = 60; g: 60<age < = 110; h: age>110. The model was fit using reduced major axis regression method. ** significant at α = 0.01 level.

Overall M_B_ was significantly related to M_A_ following a scaling function with a scaling exponent of 0.964 (Fig. 5). To investigate the influences of the biotic (i.e. tree age, size and density) and abiotic factors (i.e. latitude, longitude, and elevation) on the relationships between M_B_ and M_A_, power functions for each age, DBH, height, density, latitude, longitude, and elevation group were developed. All 56 equations (one estimate for each of eight subdivisions of the data classified by seven abiotic and biotic factors) were significant at α  = 0.05 level. For example, M_B_ scaled isometrically with M_A_ within all 8 age groups (Fig. 6). The mean value of the coefficient of determination (r^2^) for the power functions was 0.903 with a range from 0.731 to 0.968. The scaling components varied slightly among age groups. The scaling exponent was 0.964±0.0040 (Table 2). Among different groups, the scaling exponent also varied slightly from 0.900 to 1.112 with a mean value of 0.986. Twenty six out of 57 scaling exponents did not differ significantly from 1 (the value predicted by the isometric hypothesis) at α = 0.05. At α = 0.01, 37 estimates of the scaling exponent did not differ from 1.

**Table 2:** Comparison of the scaling exponent and constant estimated by different regression methods.

Group	Mean Scaling Exponent				Mean Scaling Constant			
	RMA	MAR	LSR	NLS	RMA	MAR	LSR	NLS
All data	0.964	0.963	0.999	1.005	0.274	0.275	1.428	4.592
Age	0.973	0.972	0.986	0.990	0.265	0.267	1.470	4.701
DBH	1.000	1.000	0.956	0.962	0.238	0.238	1.553	5.031
Height	0.984	0.984	0.970	0.975	0.249	0.250	1.528	4.949
Density	1.015	1.019	0.910	0.890	0.223	0.221	1.696	6.109
Latitude	0.986	0.988	0.975	0.978	0.254	0.253	1.508	4.911
Longitude	0.975	0.975	0.990	0.980	0.264	0.264	1.474	4.742
Elevation	0.965	0.964	0.998	1.008	0.273	0.275	1.430	4.491
Mean	0.986	0.986	0.969	0.969	0.252	0.252	1.523	4.991
Isometric relationship	26(46%), 37(65%)	34(60%), 42(74%)	14(24%), 24(42%)	20(35%), 30(53%)				

RMA: Reduced major axis regression; MAR: Major axis regression; LSR: Linear regression on log-log transformed data; NLS: Weighted non-linear regression. DBH: diameter at breast height. The scaling constants for RMA, MAR, and LSR were reverse transformed. For any grouping method, the exponent and constant presented here is the average of the exponent or constant estimated for each of the 8 categories in that group. Isometric relationship values are the number of times that the 95% and 99% confidence intervals of scaling exponent estimations, respectively, included 1 (followed by the percentage for each). The total number of scaling exponents estimated was 57.

**Figure 7:**
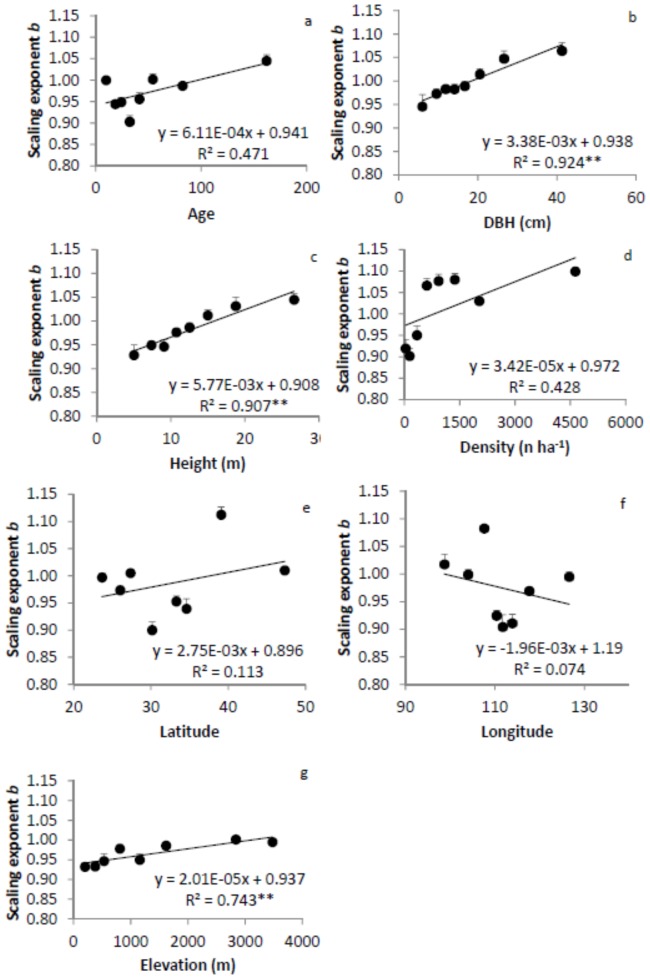
Relationships between scaling exponent and tree age (a), diameter at breast height (DBH, b), height (c), density (d), latitude (e), longitude (f), and elevation (g). The scaling exponent of each age, DBH, height, density, latitude, longitude, and elevated group was estimated using reduce major axis (RMA) regression analysis. The model with the best fit among the linear, quadratic and power function models is presented. ** significant at α = 0.01 level. Error bars are standard errors of the slopes.

The scaling exponent showed significant relationships with tree size, but not with tree age or density (Fig. 7). It increased linearly with DBH and height, and showed trends of increase with age and density. The scaling exponent also increased with elevation, but not with latitude or longitude.

### Influences of Biotic and Abiotic Factors on the Scaling Constant

**Figure 8:**
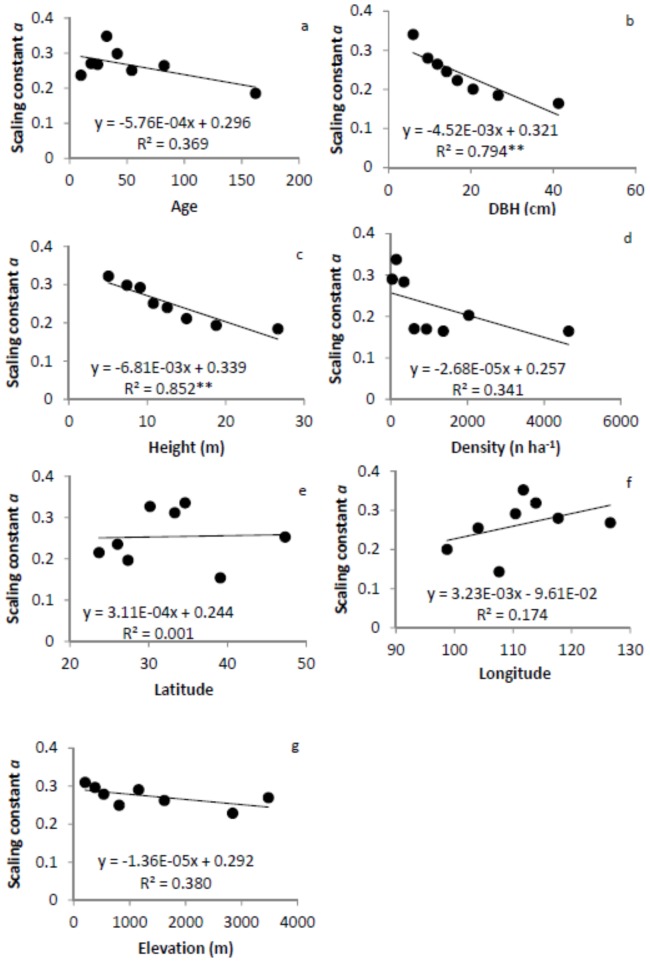
Relationships between scaling constant/intercept and tree age (a), diameter at breast height (DBH, b), height (c), density (d), latitude (e), longitude (f), and elevation (g). The model with the best fit among the linear, quadratic and power function models is presented. ** significant at α = 0.01 level. Error bars are standard errors of the scaling exponents.

The scaling constant was 0.274 for all data combined. It varied from 0.143 to 0.352 among different groups with a mean value of 0.252 for all groups. Mean scaling constant varied slightly among different groups, with the lowest value for density and largest one for elevation. The scaling constant showed significant relationships with tree size, but not with any other factors (Fig. 8). It decreased linearly with DBH and height, but did not change with latitude and longitude. There were trends of decrease in the scaling constant with tree age, density, and elevation.

### Influences of Regression Methods on the Scaling Exponent and Constant

Compared to RMA, MAR produced very similar estimates of the scaling components (Table 2). The mean value of all scaling exponents from MAR was 0.986 with 34 and 42 of the 57 scaling exponents not significantly different from 1 at α = 0.05 and 0.01 levels, respectively. LSR produced lower scaling exponents, and higher scaling constants, compared to RMA. NLR produced similar exponents when weighted NLR was used with a weighting factor of 1/M^3/4^ [40], but estimated higher scaling constants than RMA. The choice of weight factor had a large influence on the scaling exponent estimation. Overall, different regression methods produced similar scaling exponents, but LSR and NLS generated larger scaling constants than RMA and MAR.

### Influences of Phylogeny and Forest Types on Root:Shoot Ratio, the Scaling Exponent and Constant

We also separated the database into different groups based on family membership, leaf form, forest origin, and phylogenetic groups: gymnosperms versus angiosperms or dicots versus monocots. We calculated mean belowground biomass, above-ground biomass, root:shoot ratio, and estimated the allometric scaling model for each group using RMA (Table 1). Among the 15 most abundant families, there were large variations in mean belowground and aboveground biomass. Aceraceae had the lowest mean belowground biomass while Taxodiaceae had the largest. For aboveground biomass, Taxodiaceae also had the highest mean but Hamamelidaceae had the lowest mean. Root:shoot ratio varied from 0.16 in Cupressaceae to 0.29 in Ulmaceae with most values in the range of 0.19 to 0.24. Root:shoot ratio was smaller for evergreens (0.19) than deciduous (0.24) trees, but similar between natural (0.21) and planted forests (0.18), between gymnosperms (0.19) and angiosperms (0.22), and between monocots and dicots (Table 1).

For all groups, significant allometric scaling models were fit (Table 1). Of the 15 most abundant families, the scaling exponent varied slightly from 0.849 in Aceraceae to 1.033 in Cupressaceae. Only one scaling exponent was significantly different from 1. The scaling exponent for deciduous forests was smaller than that for evergreen forests and significantly different from 1. Gymnosperms had a slightly higher scaling exponent than angiosperms. Natural forests had a lower scaling exponent than 1, but planted forests had a scaling exponent close to 1.

## Discussion

We calculated root:shoot ratio and developed the allometric scaling relationship between M_B_ and M_A_ using a large database of forest biomass in China. Whether or not either the ratio or the scaling exponent and constants varied with tree age, size, density, latitude, longitude, elevation, and phylogeny was tested by subdividing the data. We found that the root:shoot ratio did not change with age, tree density, longitude or elevation, but decreased with tree size and increased with elevation (Fig. 4). Little variation in root:shoot ratio was observed among families, forest origins, and other forest type groups (Table 1). In general, M_B_ scaled isometrically with M_A_ in Chinese forest ecosystems. The scaling exponents did not vary with tree age, density, latitude, or longitude, but linearly increased with DBH, height and elevation (Fig. 7), partially supporting our hypotheses. The mean of scaling exponents of all groups was 0.986 with a small range of 0.964 to 1.015, close to the value of 1 predicted by the isometric hypothesis. Different regression methods produced similar scaling exponents, but different scaling constants. These results demonstrate that biotic and abiotic factors may have limited influence on the scaling exponent. Considering tree size and elevation, however, may improve the estimation of M_B_ from M_A_.

Root:shoot ratio has been commonly used as an indicator for the relative partition between belowground and aboveground biomass [45]–[47]. Root:shoot ratios for forest ecosystems have been estimated in several previous studies [29], [32], [48]. For example, Cairns *et al.* [29] reported that the root:shoot ratio varies from 0.05 to 0.70, with a mean value of 0.26 and the tendency of values to be between 0.20 and 0.30, using a database of 165 records collected from literature. Wang *et al.* [47] reported that the root:shoot ratio ranges between 0.09 and 0.67 with a mean of 0.27 using a database of 515 records of forest biomass in northeast China. Using a larger database of 649 paired above- and below-ground data, Luo *et al.* [49] recently reported that root:shoot ratio varied from 0.070 to 0.730, with a mean of 0.233. Our estimation of the root:shoot ratio of forests in China was 0.202±0.00087, a value toward the low end of both Wang *et al.* [47] and Luo *et al.*’s [49] ranges. In addition, there was much less variation in the ratios estimated by us compared to those estimated in the earlier studies of Chinese forests.

Our results supported our hypotheses on DBH, height and latitude influences, but not on age, longitude, and elevation. Whether the root:shoot ratio varies with age and other variables has been controversial. For example, the root:shoot ratio has been reported to decrease with stand age across forests and woodlands [32] and in northeastern China [47]. Mokany *et al.* [32] reported that the root:shoot ratio was negatively related to forest stand age, height, shoot biomass, precipitation, and temperature. The ratio did not vary with tree age in our study (Fig. 4), which disagrees with Mokany *et al.*’s [32] results, but agrees with the results reported by Cairns *et al.* [29] and Yang & Luo [14]. Cairns *et al.* [29] found that the ratio does not vary with tree age, type, temperature, or precipitation. The constancy of the root:shoot ratio in forests of different age has been attributed to different biomass partitioning patterns among individual tree species and nutrient availability changes over the normal age sequence [14]. Luo *et al.* [49] reported significant yet weak negative relationships of root:shoot ratio with mean annual temperature and precipitation (r^2^ = 0.08 and 0.13, respectively). Our results also showed that the root:shoot ratio initially declined with DBH and height, which supports the findings by Mokany *et al.* [32] and Wang *et al.* [47]. The relationships of root:shoot ratio with DBH or height that they developed are significant but weak. Using binned data in this study, we revealed clearer patterns (Fig. 4). The decline of the root:shoot ratio with DBH and height might be a result of plant ontogeny, related to the accumulation of M_A_ in woody tissue as stands develop [32], [47]. The root:shoot ratio also increased with latitude, as trees allocated more carbon to the belowground in the cold and dry high latitude regions than in the warm and wet low latitude regions.

Another finding of this study was that M_B_ scaled isometrically or near isometrically with M_A_ in Chinese forest ecosystems, consistent with the prediction of the isometric hypothesis. The mean of scaling exponents of all groups was 0.986 with a small range of 0.964 to 1.015, close to 1 predicted by the isometric hypothesis. The 95% confidence intervals for scaling exponents included 1 in 26 out of 57 relationships for the 95% confidence intervals and in 37 out of 57 instances for the 99% confidence intervals, and did not vary with tree age, density, latitude, or longitude. Our results were consistent with many previous studies which considered different forest ecosystems [12], [14], [21], [26].

In the absence of physiological measurements, explanations of these results may seem to be speculative. But our hypotheses may promote a mechanistic understanding of biomass partitioning and influence the design of future experimental studies. One hypothesis to explain the invariant scaling relationship is that of a developmental constraint [50]–[52]. The developments of different organs are correlated and might limit the ability of each organ to develop an independent response [20], [51]. Size correlation among different organs may also prohibit plants to respond independently to environmental variation [52]. Plants may allocate proportionally their annual growth aboveground and belowground with increasing age and development [21], [41] and the partitioning does not have a significant pattern with geographical locations.

The near isometric relationship between M_B_ and M_A_ may also be linked to nutrient and water uptake [46], [53]–[54]. The limitation of water and nutrient uptake by the roots may limit the growth of aboveground biomass. Gross differences in water availability may be more regulated by stomatal responses than by adjustments in biomass allocation [53]. Decline in soil moisture might have similar effects on plant photosynthesis and nutrient uptake and result in no allocation adjustment. Over the long term, interannual variation in precipitation and soil water availability may also even out the imbalance of growth of belowground and aboveground and biomass allocation, as root biomass may be enhanced more in one year while aboveground biomass is enhanced in another year.

Statistically significant variation may also exist in the scaling exponent, depending on how the data are sorted into different categories [12]. The scaling exponents indeed increased linearly with DBH, height and elevation (Fig. 7). The different relationships of the scaling exponent with age and size seem to be contradictory to each other. However, the difference resulted from the fact that trees of the same age of different species were different sizes, so size and age were not closely correlated. Age may not be a good indicator for species development in mixed species forests. Tree size (DBH and height) displayed significant influences on the scaling exponent. The scaling exponent increased with both DBH and height, indicating that a relatively larger portion of photosynthate was allocated to belowground biomass in larger trees than in smaller ones. As for the elevation, trees growing in the eastern lower elevation regions tend to be smaller than those in the higher elevation regions, partially due to more frequent harvesting in the lower elevation regions. The scaling exponent increased with elevation. We also showed that different regression methods had little influence on the scaling exponent, a conclusion that had been reached earlier in a study when the correlation coefficient was large (e.g., r>0.90) [43].

Whereas the scaling exponent of the scaling relationship is considered as the results of universal physical constraints and natural selection, the scaling constant of the relationship has been linked to various taxon-specific or ecological factors [8], [21], [24], [55]–[57]. Cheng *et al.* [58] reported that the scaling constant decreases with stand age in northern Chinese forests. This study involved a systematic analysis of both biotic and abiotic factors. Here, we found that the scaling constant varied with tree size, but not with tree age, density, latitude, longitude, or elevation (Fig. 8). Smaller trees had higher scaling constants than larger trees, indicating that if the scaling exponents are the same, smaller trees would allocate more carbon belowground. It is worthy of note that the decreases in the scaling constant with DBH and height more than offset the positive effect of increases in the scaling exponent, resulting in overall decreases in root:shoot ratio with DBH and height.

Our analyses of the influence of phylogeny and forest type on the scaling relationship confirmed that M_B_ scaled isometrically with M_A_ in 14 of the 15 most abundant families, most phylogeny groups (e.g., gymnosperms, dicots and monocots), leaf forms, and forest origins (i.e. natural and plantation). These results were comparable to Luo *et al.* [49] who reported that the scaling exponent varied from 0.763 to 1.047 among 17 forest species groups. These results indicated that widely different plant species are convergent with regard to the allometry of biomass partitioning [9], [20], [21]. While some individual scaling exponents were significantly different from 1, those that differed were still close to 1 and our results generally support the isometric hypothesis.

As more ecological data accumulate, data synthesis becomes an important tool to reveal general patterns and ecological rules [8], [11], [59]. As in any data synthesis, one must be aware of certain limitations when interpreting these results. As summarized by Mokany *et al.* [32], there are several methodological pitfalls associated with sampling root biomass in forests. These include sampling too shallow to capture the majority of roots, lacking of sampling the root crown of woody plants, and sampling with small number of replications that resulted in an unreliable estimate of root biomass [32]. Coarse root biomass estimations of shallow root species such as spruce and fir were usually more accurate than those of deep root species. Due to these reasons, M_B_ could be underestimated and result in a lower root:shoot ratio. Slightly different sampling methods in individual studies collected in the database might also add biases to biomass estimation. Despite this, it is evident that M_B_ is closely related to M_A_ in Chinese forests. More accurate estimation in M_B_ could improve our understanding of root biomass and biomass partitioning. Nevertheless, the relationship developed in this study could be very helpful for the estimations of root biomass and ecosystem carbon dynamics in forests. The root:shoot ratios and allometric scaling models for different families, phylogeny groups and forest types could be used in ecological modeling. Considering the tree size and other factors could provide more accurate estimations in forest biomass and reduce the uncertainty at regional scales.

## Conclusions

Using a large forest biomass database, we developed the allometric scaling relationships between M_B_ and M_A_ and tested systematically how biotic and abiotic factors would influence their relationship. We demonstrated that the scaling component did not vary with tree age, density, latitude or longitude, but varied with tree size and elevation. The overall small variations of scaling exponent within each group and among different groups indicated that M_B_ scaled isometrically or near isometrically with M_A_. While fitting a single allometric scaling relationship may be adequate, the estimation of M_B_ from M_A_ could be improved with size-specific scaling relationships.
